# A Basic Guide to the Growth and Manipulation of the Blast Fungus, *Magnaporthe oryzae*

**DOI:** 10.1002/cpz1.523

**Published:** 2022-08-23

**Authors:** Camilla Molinari, Nicholas J. Talbot

**Affiliations:** ^1^ The Sainsbury Laboratory, Norwich Research Park University of East Anglia Norwich United Kingdom

**Keywords:** appressorium, conidia, culture, fungal development, *M. oryzae*, mycelium, *Pyricularia*, virulence

## Abstract

The blast fungus, *Magnaporthe oryzae*, is a devastating plant pathogen that threatens global food security. The social and economic importance of blast disease has contributed to this filamentous fungus becoming a model organism for the study of host‐pathogen interactions. Availability of the complete genome sequences of many strains of the pathogen, as well as rice and wheat cultivars, coupled with the tractability of *M. oryzae* to classical and molecular genetic manipulation have contributed to its widespread study. Although *M. oryzae* has been extensively investigated for the past two decades, procedures for storing, maintaining, and manipulating the blast fungus in the laboratory had not been compiled and updated. As a consequence, there is considerable disparity in how the fungus is stored and manipulated between studies. In this article, we present a collection of protocols providing clear explanations of how to preserve filter stocks of *M. oryzae*; how to grow the fungus in both liquid and solid media; how to extract genomic DNA from fungal mycelium; how to induce appressorium formation on coverslips for visualization and tissue collection; and how to perform two distinct types of plant infection assay for virulence assessment. By sharing our most used laboratory procedures, we aim to address some of the knowledge gaps in current *M. oryzae* protocols and contribute to uniformity and robustness in studies by the *Magnaporthe* research community. © 2022 The Authors. Current Protocols published by Wiley Periodicals LLC.

**Basic Protocol 1**: Storage of *M. oryzae* strains

**Basic Protocol 2**: Revival and regular maintenance of *M. oryzae* cultures in solid medium

**Alternate Protocol 1**: Regular maintenance of *M. oryzae* cultures in liquid medium

**Basic Protocol 3**: Genomic DNA extraction from *M. oryzae* mycelium

**Alternate Protocol 2**: Quick DNA extraction from *M. oryzae* mycelium

**Basic Protocol 4**: *M. oryzae* induction of appressorium development on glass coverslips for microscopy

**Alternate Protocol 3**: *M. oryzae* induction of appressorium development on glass coverslips for tissue collection

**Basic Protocol 5**: *M. oryzae* rice infection assay through spray inoculation

**Alternate Protocol 4**: *M. oryzae* leaf‐drop plant infection assay

## INTRODUCTION

*Magnaporthe oryzae* (synonym of *Pyricularia oryzae* Sacc.) is a haploid ascomycete fungus best known as the causal agent of rice blast disease (Talbot, 2003; Valent, Farrall, & Chumley, 1991; Zhang et al., 2016). The blast fungus has been reported to infect over 50 different grass species, including *Setaria*, *Eragrostis*, *Panicum*, *Leersia*, and *Lolium* species, but individual host‐limited forms are normally restricted to particular host species. *M. oryzae* causes important diseases of rice (*Oryza sativa*), barley (*Hordeum vulgare*), wheat (*Triticum aestivum*), and finger millet (*Eleusine coracana*) (Wilson & Talbot, 2009). The agricultural and economic damage caused by *M. oryzae* globally has stimulated extensive research on the fungus and its hosts, which are now established models for the study of fungal pathogenesis and plant immunity. The availability of the complete genome sequences of many isolates of *M. oryzae* and its hosts, such as rice and wheat (Brenchley et al., 2012; Dean et al., 2005; Yano et al., 2016), and the amenability of *M. oryzae* to genetic manipulation have provided strong reasons for its adoption as a study organism. As a result, there is now a growing understanding of the cell and developmental biology of blast disease (Eseola et al., 2021; Fernandez & Orth, 2018; Talbot, 2003; Valent & Chumley, 1991; Wilson & Talbot, 2009). Future investigation is, however, imperative to develop new strategies with which to successfully control *M. oryzae*, and the adoption of common procedures is critical to ensure reproducibility and robustness of global research on blast diseases.

To cause disease, *M. oryzae* develops a specialized infection cell called an appressorium to gain entry to host tissue (Mendgen, Hahn, & Deising, 1996). Appressoria are a common mechanism deployed by diverse fungal pathogens to invade their hosts (Talbot, 2019). *M. oryzae* appressoria generate enormous turgor, which is focused as physical force to rupture the leaf cuticle, thereby facilitating entry to host internal tissues (Talbot, 2019). Appressoria have been extensively studied in *M. oryzae* because they represent the developmental stage most important to achieving infection (Dean et al., 2012; Howard & Valent, 1996). Early development of appressoria starts with the germination of a three‐celled conidium after it lands on the hydrophobic leaf surface (Hamer, Howard, Chumley, & Valent, 1988). The apical cell of the conidium germinates to form a narrow germ tube that grows across the leaf before differentiating at its tip to develop a dome‐shaped appressorium (Talbot, 2003). Turgor in the appressorium is generated by glycerol accumulation in the cell, which draws in water by osmosis. The appressorium is lined with melanin in its cell wall, which reduces wall porosity, enabling turgor to be maintained (de Jong, McCormack, Smirnoff, & Talbot, 1997; Talbot, 2003). Once a critical threshold of turgor has been generated in the appressorium (Ryder et al., 2019), penetration peg development occurs; this involves a reorientation of the actin cytoskeleton to form a toroidal network at the base of the infection cell, which is scaffolded and maintained through the action of fungal septins (Dagdas et al., 2012). The fungus then rapidly colonizes rice tissue using invasive hyphae, enveloped by the host plasma membrane. Invasive hyphae move between plant cells using pit field sites where plasmodesmata accumulate and continually move into living plant cells. At the same time, the fungus secretes an extensive battery of fungal effectors to suppress plant immunity. In this way, *M. oryzae* overwhelms its host to bring about disease symptoms within 72 hr of initial infection, producing necrotic lesions that spread and coalesce. *M. oryzae* then sporulates from disease lesions, producing conidia for several weeks; these can each infect new host plants, being spread by wind and dewdrop splash. In this way, blast epidemics, particularly in rice, can spread rapidly and cause extensive damage. Rice blast is one of the most chronic and widespread diseases of rice (Savary et al., 2019).

Methods for the storage and culture of *M. oryzae* have been developed in many laboratories around the world but have not been standardized or collated. Procedures for appressorium induction on artificial surfaces in the laboratory, and rice blast infection assays have also been developed, but have not previously been described in comprehensive detail. A compilation of basic guidance on how to work with the blast fungus is therefore imperative to address many gaps in these procedures that may be easily overlooked by new researchers entering the field. Additionally, sharing of protocols will lead to greater accuracy and reproducibility, which are major prerequisites to achieving result uniformity within the blast disease research community. In this article, we describe the key steps for working with the blast fungus. In each protocol, the overriding aim is to reproduce a specific stage of the *M. oryzae* life cycle from its natural environment under laboratory conditions. We describe methods for preservation of *M. oryzae* strains, growth of the fungus in culture to generate mycelium and conidia, induction of appressorium development, and infection assays performed on rice and barley hosts. Additionally, we include a robust protocol for extracting genomic DNA from fungal mycelium in order to carry out genetic manipulations and genotypic characterization.

*CAUTION: Magnaporthe oryzae* is a Biosafety Level 1 pathogen; follow all appropriate guidelines and regulations for the use and handling of pathogenic microorganisms. It is also advisable to use UV light for decontamination before and after working with this organism.

*NOTE*: To avoid contamination of the cultures, follow strict aseptic technique and work in a circulating airflow cabinet (class II) when handling *M. oryzae*.

## Basic Protocol 1 STORAGE OF *M. ORYZAE* STRAINS

Filamentous fungi can be effectively stored by growing mycelium through dry filter paper (Fong, Anuar, Lim, Tham, & Sanderson, 2000; Gupta et al., 2020; Jia, 2009). This is a simple, inexpensive method to preserve fungal mycelium for many years. *M. oryzae* overwinters in rice straw in the form of fungal mycelium. In contrast to many fungal species, conidia of *M. oryzae* are not dormant structures and are not long lived; their primary role is to germinate rapidly and produce appressoria (Talbot, 2019). The fungus therefore uses mycelium within infected plant tissue as its dormant state. Infected rice straw provides a dry environment in which the fungus survives the winter. Following rainfall in the spring, *M. oryzae* sporulates from straw material and conidia then infect new plants. This protocol mimics this stage of the life cycle of the fungus, using filter paper as an environment in which the fungal mycelium can survive for long periods, once desiccated. Viability of fungal mycelium from desiccated filter stocks preserved using this protocol has shown to still recover perfectly after 30 years at −20°C, demonstrating the efficiency of the technique.

*CAUTION: Magnaporthe oryzae* is a Biosafety Level 1 pathogen; follow all appropriate guidelines and regulations for the use and handling of pathogenic microorganisms.

### Materials

70% (v/v) ethanol (in water) for decontaminating materials and equipment
Viable *M. oryzae* strains (e.g., Guy11, the most commonly used rice blast strain, available from the corresponding author's laboratory)
Complete medium (CM) sterile plates (see recipe)

Circulating Class II flow hood (BSC Class II or equivalent)
Stainless‐steel surgical scalpel handle and scalpel (e.g., Fisher Scientific cat. no. 11890862 and 12961645)
Stainless‐steel forceps (e.g., Fisher Scientific Nickel Electro™ cat. no. 15699310)
Whatman 3MM filter paper cut into ∼2‐mm^2^ squares (e.g., Fisherbrand™ cat. no. 09800)
Incubator with timer (e.g., Fisher Scientific cat. no. 15500214), set at 24°C with a 12‐hr photoperiod
Bunsen burner (e.g., Fisher Scientific cat. no. 03391301)
60 × 15‐mm disposable polystyrene petri dishes (e.g., Sigma‐Aldrich cat. no. P5481)
Micropore surgical paper tape (e.g., Fisher Scientific cat. no. 12787597)
Vacuum desiccator
Lidded plastic/Tupperware container (e.g., Lakeland lidded box food storage 2.5 L)
2‐ to 5‐mm orange indicator silica gel desiccant beads (e.g., Trustleaf cat. no. SK‐9304226)
30 × 50‐mm paper bags (e.g., Fisher Scientific cat. no. 2152101), autoclaved
Freezer (e.g., Fisher Scientific cat. no. TSX2320FA), set at −20°C

**Figure 1:**
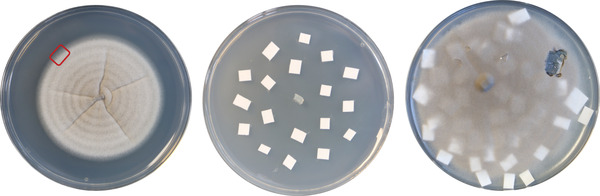
Left, a 7‐day‐old plate culture of *M. oryzae* (Guy11) after incubation at 24°C with a 12‐hr photoperiod. The red square indicates the growth margin where the mycelium should be cut to be transferred onto fresh CM agar for subculture. Middle, a CM medium agar plate with a piece of *M. oryzae* mycelium inoculated in the center and Whatman filter paper squares for the generation of fungal filter stocks. Right, a 12‐day‐old *M. oryzae* culture grown on CM agar with filter papers through which fungal mycelium has grown (at top right, a filter through which mycelium has grown has been collected from the plate).

1 Using a steel scalpel, cut a 1‐ to 5‐mm^2^ piece of mycelium from the growing margin of a 7‐ to 10‐day‐old *M. oryzae* culture (as indicated in Fig. 1) and transfer to a fresh CM agar plate.
  Work should be performed in a circulating Class II flow hood near a Bunsen burner. Remember to sterilize scalpel before and after it is in contact with the fungus following proper aseptic techniques.

2 Using steel forceps, place 20‐30 pieces of Whatman filter paper, cut into ∼2‐mm^2^ squares, around the transferred piece of mycelium on the CM agar plate (as indicated in Fig. 1).
  The size of the cut pieces of Whatman filter paper can vary, but smaller pieces are recommended to allow the fungus to grow through them successfully. When generating filter stocks, CM agar is usually not supplemented with any antibiotics, allowing the fungus to grow in optimal conditions before storage.
3 Incubate plates at 24°C with a 12‐hr photoperiod until mycelium has grown through all of the filter papers.
  Do not leave plates incubating for >2 weeks before collecting the filter papers. Leaving plates for too long can reduce viability of the strain during storage.
4 Using flame‐sterilized steel forceps, collect the filter papers covered with *M. oryzae* mycelium, place these in 60 × 15‐mm vented petri dishes, and seal them with micropore surgical paper tape.
  Work should be performed in a circulating Class II flow hood near a Bunsen burner. Remember to sterilize forceps before and after they are in contact with the fungus following proper aseptic techniques.
5 Place petri dishes in a vacuum desiccator for 48 hr to dry fully. Alternatively, if a vacuum desiccator is not available, the petri dishes containing filters can be placed in a lidded plastic container containing a 1‐ to 2‐cm depth of silica gel desiccant and left to desiccate for 10‐15 days at room temperature.
  Make sure the plastic/Tupperware container is in a dry place, using fresh silica gel each time to enable complete desiccation of the filter papers. Freezing humid filter papers greatly diminishes the viability of the fungal culture stock.
6 Once the filter papers are completely desiccated, transfer them to autoclaved flat paper bags using sterilized steel forceps.
  Work should be performed in a circulating Class II flow hood near a Bunsen burner. Remember to sterilize forceps before and after they are contact with the fungus following proper aseptic techniques.
7 Filter bags containing the fungal stocks are stored at −20°C inside a lidded plastic/Tupperware container with 1‐2 cm^3^ of silica gel.
  If kept properly, M. oryzae filter stocks can be stored for many years. Currently, in our laboratory, we still revive 30‐year‐old filter stocks successfully.

## Basic Protocol 2 REVIVAL AND REGULAR MAINTENANCE OF *M. ORYZAE* CULTURES IN SOLID MEDIUM

*M. oryzae* is commonly cultured in solid CM (complete medium) agar plates. The strain can be grown directly by placing a frozen filter paper stock on a plate or by transferring a 2‐ to 5‐mm piece of mycelium of another growing culture onto fresh growth medium. Every transfer must be done carried out in a circulating Class II flow hood under sterile conditions.

*CAUTION: Magnaporthe oryzae* is a Biosafety Level 1 pathogen; follow all appropriate guidelines and regulations for the use and handling of pathogenic microorganisms.

### Materials

70% (v/v) ethanol (in water) for decontaminating materials and equipment
Viable *M. oryzae* filter frozen stocks (e.g., Guy11; see Basic Protocol 1)
Complete medium (CM) sterile plates (see recipe), with or without antibiotics (see step 1)

Circulating Class II flow hood (BSC Class II or equivalent)
Stainless‐steel forceps (e.g., Fisher Scientific Nickel Electro™ cat. no. 15699310)
Incubator with timer (e.g., Fisher Scientific cat. no. 15500214), set at 24°C with a 12‐hr photoperiod
Stainless‐steel surgical scalpel handle and scalpel (e.g., Fisher Scientific cat. no. 11890862 and 12961645)
Bunsen burner (e.g., Fisher Scientific cat. no. 03391301)

1 Using steel forceps, pick one *M. oryzae* desiccated filter stock (directly from frozen at −20°C) and place it on the middle of a CM agar plate. Alternatively, a small fragment of a filter stock paper is easily sufficient and can be cut from a stock paper using flame‐sterilized scissors.
  Work should be performed in a circulating Class II flow hood near a Bunsen burner. Remember to sterilize forceps and scissors before and after they are in contact with the fungus following proper aseptic techniques. Use appropriate antibiotics to prevent bacterial contamination, if necessary, but this is not advised for routine subculture. If required, stock filter papers can be cut and separated to numerous CM agar plates, as 1 mm^2^ of desiccated filter containing M. oryzae will provide sufficient mycelium to revive on CM medium.
  Routinely used antibiotics include penicillin‐streptomycin (50 U/ml; e.g., Fisher Scientific cat. no. 15140122) for prevention of bacterial contamination. For selection of fungal transformants, hygromycin B (200 μg/ml; e.g., Fisher Scientific cat. no. 10687010), BASTA (glufosinate ammonium; 100 μg/ml; e.g., Sigma‐Aldrich cat. no. G4670), and sulfonylurea (chlorimuron ethyl; 100 μg/ml; e.g., Sigma‐Aldrich cat. no. 06846) are commonly used for M. oryzae.
2 Incubate plates 7‐10 days at 24°C with a 12‐hr photoperiod to obtain a 5‐ to 7‐cm *M. oryzae* culture.
  Use 7‐ to 10‐day‐old colonies, and never older than 14 days, to avoid viability issues.
3 Using a steel scalpel, cut a 1‐ to 5‐mm^2^ piece of mycelium from the growing margin of the 7‐ to 10‐day‐old *M. oryzae* culture (as indicated in Fig. 1a) and transfer to a fresh CM agar plate.
  Work should be performed in a circulating Class II flow hood near a Bunsen burner. Remember to sterilize the scalpel before and after it is in contact with the fungus following proper aseptic techniques. Use appropriate antibiotics to prevent contamination.
4 Incubate plates 7‐10 days at 24°C with a 12‐hr photoperiod to obtain a 5‐ to 7‐cm *M. oryzae* culture.
  Use the colony as soon as possible once grown and when no older than 2 weeks. Culture transfer for successive use should only be performed once because original viability and phenotypes can be lost, which is why reviving M. oryzae from filter stocks is always recommended.

## Alternate Protocol 1 REGULAR MAINTENANCE OF *M. ORYZAE* CULTURES IN LIQUID MEDIUM

For many manipulations, *M. oryzae* will need to be grown in liquid CM broth. This type of mycelial growth is fast, and 24‐48 hr of incubation generates large amounts of fungal mycelium that can be used to extract genomic DNA, total RNA, and protein from the fungal tissue. Furthermore, fungal protoplasts for *M. oryzae* transformation are also generated from fugal mycelium grown in liquid culture.

*CAUTION: Magnaporthe oryzae* is a Biosafety Level 1 pathogen; follow all appropriate guidelines and regulations for the use and handling of pathogenic microorganisms.

### Materials

Viable *M. oryzae* filter frozen stocks (see Basic Protocol 1)
70% (v/v) ethanol (in water) for decontaminating materials and equipment
Complete medium (CM) sterile plates (see recipe)
Liquid CM (see recipe)

Circulating Class II flow hood (BSC Class II or equivalent)
Stainless‐steel surgical scalpel handle and scalpel (e.g., Fisher Scientific cat. no. 11890862 and 12961645)
Stainless‐steel forceps (e.g., Fisher Scientific Nickel Electro™ cat. no. 15699310)
Bunsen burner (e.g., Fisher Scientific cat. no. 03391301)
Stainless steel blender container (e.g., Container for Waring® Blenders, 360 ml, cat. no. 58983‐004)
Two‐speed 1‐liter laboratory blender (e.g., Waring® cat. no. WZ‐04242‐11)
250‐ml glass Erlenmeyer flask (e.g., Fisher Scientific cat. no. 15436143)
Polyurethane foam stopper (e.g., Fisher Scientific cat. no. 12984291)
Aluminum foil (e.g., Lakeland foil)
Rotary shaking platform (e.g., Fisher Scientific cat. no. 11676076)
Incubator with timer (e.g., Fisher Scientific cat. no. 15500214), set at 24°C with a 12‐hr photoperiod

**Figure 2:**
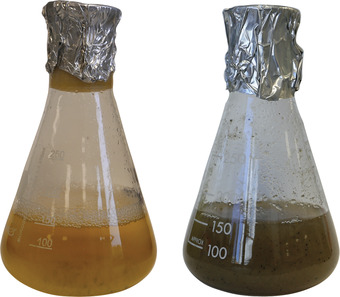
*M. oryzae* liquid culture of strain Guy11 in liquid CM medium before (left) and after (right) incubation for 30 hr at 24°C with a 12‐hr photoperiod in a rotary shaker set at 100 rpm. A typical liquid culture is shown, made using a 250‐ml Erlenmeyer flask covered with a foam stopper and aluminum foil to prevent contamination.

1 Using a steel scalpel, cut all the mycelium from the growing margin of a 7‐ to 10‐day‐old *M. oryzae* culture and transfer it to a stainless‐steel container for blending. Add 250 ml liquid CM.
  Work should be performed in a circulating Class II flow hood near a Bunsen burner. Remember to sterilize scalpels before and after they are in contact with the fungus following proper aseptic techniques. Include appropriate antibiotics in the liquid CM only when necessary to prevent contamination (penicillin‐streptomycin, 50 U/ml; e.g., Fisher Scientific cat. no. 15140122). Steel blending containers must be autoclaved before use.
2 Blend the solid inoculum with the liquid CM in a two‐speed blender, 5 s at the first speed followed by 10 s at the second speed.
  Do not remove blender lid outside a circulating Class II flow hood to prevent contamination of the culture.
3 Transfer the blended culture into an autoclaved 250‐ml glass Erlenmeyer and cover with a foam stopper and sterilized aluminum foil, as indicated in Figure 2.
  Transfer must be done in a circulating Class II flow hood.

4 Incubate the flask on a rotary shaker set at 100 rpm in an incubator at 24°C with a 12‐hr photoperiod for 30‐48 hr.
  Usually, the liquid culture will turn a light brown color as shown in Figure 2. However, if the culture is overgrown, it will turn a darker color, indicating that it is too old and overgrown for experimentation.

## Basic Protocol 3 GENOMIC DNA EXTRACTION FROM *M. ORYZAE* MYCELIUM

*M. oryzae* genomic DNA extraction can be performed starting from fungal mycelium grown on liquid or solid agar medium. This is a multi‐step protocol aiming to obtain purified fungal DNA free from proteins, polyphenols, tannins, and polysaccharides. After several years of refining DNA extraction methods, we have found that a cetyl trimethyl ammonium bromide (CTAB) protocol works successfully for extraction of high‐quality plant, bacteria, yeast, and fungal DNA (Rogers & Bendich, 1994; Saghai‐Maroof, Soliman, Jorgensen, & Allard, 1984). This protocol, which has been updated and modified, works very well for *M. oryzae* genomic DNA extraction.

*CAUTION: Magnaporthe oryzae* is a Biosafety Level 1 pathogen; follow all appropriate guidelines and regulations for the use and handling of pathogenic microorganisms.

### Materials

70% (v/v) ethanol (in water) for decontaminating materials and equipment
7‐ to 10‐day‐old *M. oryzae* fungal culture grown on CM agar medium (e.g., Basic Protocol 2 or Alternate Protocol 1)
Complete medium (CM) with agar (see recipe)
CTAB buffer (see recipe), prewarmed to 65°C
24:1 (v/v) chloroform/isoamyl alcohol (CIA)
Isopropanol, prechilled to −20°C
TE buffer with RNase (see recipe)
3 M NaOAc, pH 5.2 (see recipe)
100% ethanol, prechilled to −20°C
Sterile distilled water

Circulating Class II flow hood (BSC Class II or equivalent)
Bunsen burner (e.g., Fisher Scientific cat. no. 03391301)
Stainless‐steel surgical scalpel handle and scalpel (e.g., Fisher Scientific cat. no. 11890862 and 12961645)
Stainless‐steel laboratory spatula (e.g., Fisher Scientific cat. no. 11523482)
Precut 5‐cm cellophane discs, sterilized in distilled water by autoclaving
Porcelain pestle and mortar (e.g., Fisher Scientific cat. no. 11740269 and cat. no. 10443471), prechilled in liquid nitrogen
Spatula, prechilled in liquid nitrogen
Incubator with timer (e.g., Fisher Scientific cat. no. 15500214), set at 24°C with a 12‐hr photoperiod
Liquid nitrogen
1.5‐ and 2‐ml microcentrifuge tubes (e.g., Eppendorf ® Microtube capacity 1.5 ml cat. no. EP0030125150 and capacity 2 ml BRAND® microcentrifuge tube cat. no. Z628034)
Single‐channel micropipets, volume 20‐200 μl and volume 100‐1000 μl (e.g., Eppendorf cat. nos. EP3123000241 and EP3123000250)
Filter tips, volume 20‐200 μl and volume 100‐1000 μl (e.g., Eppendorf cat. nos. EP3123000055 and EP3123000063)
Paper towel (e.g., DIY)
Water bath (e.g., Fisher Scientific cat. no. TSCOL19), set at 65°C
High‐speed microtube shaker (e.g., Fisher Scientific cat. no. 02217737)
Microtube centrifuge (e.g., Fisher Scientific cat. no. 75002435)
Freezer (e.g., Fisher Scientific cat. no. TSX2320FA), set at −20°C

1 Using a steel scalpel, cut a 1‐ to 5‐mm^2^ piece of mycelium from the growing margin of a 7‐ to 10‐day‐old *M. oryzae* culture (as indicated in Fig. 1) and transfer it to a fresh CM agar plate containing a precut and sterilized 5‐cm cellophane disc. Incubate at 24°C with a 12‐hr photoperiod for 7‐10 days, until fungal mycelium has grown over the edge of the cellophane.
  Work should be performed in a circulating Class II flow hood near a Bunsen burner. Remember to sterilize the scalpel before and after it is in contact with the fungus following proper aseptic techniques. Mycelium plug should be placed on top of the cellophane.
2 The fungus will grow over the cellophane, covering it completely. Once grown, transfer using sterilized forceps the cellophane with fungal mycelium onto a prechilled pestle and mortar containing liquid nitrogen for grinding. Grind vigorously to obtain a fine light‐brown powder.
  CAUTION: Use appropriate personal protective equipment when handling liquid nitrogen.
  Do not overgrow culture, as eventually the fungus will disintegrate the cellophane, making it impossible to collect. Do not allow fungal tissue to defrost while grinding or transferring to microcentrifuge tube. Either before or after grinding, the sample can be frozen and stored at −80°C for future use.
3 With a prechilled spatula, transfer the powder to a 2‐ml microcentrifuge tube, filling it to two‐thirds of the total capacity.
  Do not fill tube to more than two‐thirds of capacity, as larger volumes will be difficult to mix with CTAB buffer, and this will reduce the efficiency of the extraction.
4 Add 600 μl prewarmed CTAB and incubate sample 30 min at 65°C on a shaking platform set to 100 rpm.
  Preheating CTAB to 65°C facilitates the mixing of fungal material with the buffer.
5 Once mixed, add 1 vol CIA (24:1 chloroform/isoamyl alcohol) and incubate 30 min at room temperature on a shaking platform set to 400 rpm.
  Usually ∼600 μl CIA is added. Perform all steps involving CIA in a fume hood.
6 Centrifuge the sample 20 min at 14,000 × *g*, room temperature, to separate the aqueous phase of the sample.
  The aqueous phase will be on top, and it is transparent.
7 Transfer the aqueous phase (supernatant) of the sample to a fresh 1.5‐ml microcentrifuge tube, add 1 vol CIA, and incubate 20 min at room temperature on a shaking platform set to 400 rpm.
  Usually ∼600 μl CIA is added.
8 Centrifuge sample at 14,000 × *g* for 10 min at room temperature.
  Can also be centrifuged chilled (4°C) up to 1 hr.
9 Transfer the supernatant to a fresh 1.5‐ml microcentrifuge tube, add 1 vol prechilled isopropanol, and leave to precipitate in ice for 5 min or for longer (1‐24 hr) in a −20°C freezer.
  Longer precipitation is recommended to obtain higher concentrations of DNA.
10 Centrifuge the sample 10 min at 14,000 × *g*, room temperature, to recover the DNA.
11 Discard the supernatant and drain the tube inverted on a paper towel for 10 min.
  Longer draining might be needed if there is still liquid inside the tube.
12 Resuspend pellet with 500 μl of TE buffer containing RNase and mix gently until pellet is no longer visible. Let stand for 30 min at room temperature or for 10 min at 37°C. Then add 50 μl of 3 M NaOAc (pH 5.2) and 1000 μl prechilled 100% ethanol, mix gently, and incubate sample in a −20°C freezer for at least 30 min.
  Sample might take longer to resuspend than 10 min/30 min, but never vortex sample. Longer precipitation is recommended to get higher concentrations of DNA.
13 Centrifuge the sample 20 min at 14,000 × *g*, room temperature, to recover the DNA.
14 Decant the supernatant, and drain upside down on a paper towel for 5 min.
15 Wash DNA by adding 400 μl of 70% ethanol and gently mixing.
  Do not resuspend pellet, just gently wash it.
16 Decant the supernatant, and drain upside down on a paper towel for 20 min.
  Make sure there is no ethanol remaining inside the tube; longer draining might be needed.
17 Resuspend the pellet with 50‐200 μl sterile distilled water.
  Volume of water used will depend on the size of the pellet of DNA to resuspend and the desired concentration. Concentration is usually measured using a NanoDrop spectrophotometer and then the samples stored at −20°C.

## Alternate Protocol 2 QUICK DNA EXTRACTION FROM *M. ORYZAE* MYCELIUM

It is also possible to extract genomic DNA from *M. oryzae* mycelium using a simple, fast protocol that can be performed in <1 hr. This protocol is commonly used to extract slightly lower‐quality DNA that can nevertheless be routinely used for genotypic characterization of *M. oryzae* strains performed by PCR.

*CAUTION: Magnaporthe oryzae* is a Biosafety Level 1 pathogen; follow all appropriate guidelines and regulations for the use and handling of pathogenic microorganisms.

### Materials

70% (v/v) ethanol (in water) for decontaminating materials and equipment
7‐ to 10‐day‐old *M. oryzae* fungal culture grown on CM agar medium (Basic Protocol 2 or Alternate Protocol 1)
TE buffer with RNase (see recipe)
Complete medium (CM) with agar (see recipe)

Circulating Class II flow hood (BSC Class II or equivalent)
Bunsen burner (e.g., Fisher Scientific cat. no. 03391301)
Stainless‐steel surgical scalpel handle and scalpel (e.g., Fisher Scientific cat. nos. 11890862 and 12961645)
1.5‐ and 2‐ml microcentrifuge tubes (e.g., Eppendorf ® Microtube capacity 1.5 ml cat. no. EP0030125150 and capacity 2 ml BRAND® microcentrifuge tube cat. no. Z628034)
Homogenizer metal beads (e.g., Fisherbrand™ cat. no. 15340158), sterilized in 70% ethanol
Single‐channel micropipet, volume 100‐1000 μl (e.g., Eppendorf cat. no. EP3123000250)
Filter tips, volume 100‐1000 μl (e.g., Eppendorf EP3123000063)
Microtube centrifuge (e.g., Fisher Scientific cat. no. 75002435)
Geno/Grinder (e.g., SPEX™ Sample Prep 2010 cat. no. 2010115)
Freezer (e.g., Fisher Scientific cat. no. TSX2320FA), set at −20°C

1 Using a steel scalpel, cut a 1‐ to 5‐mm^2^ piece of mycelium from the growing margin of a 7‐ to 10‐day‐old *M. oryzae* culture (as indicated in Fig. 1a) and transfer it to a 2‐ml microcentrifuge tube containing one sterile homogenizer metal bead.
  Work should be performed in a circulating Class II flow hood near a Bunsen burner. Remember to sterilize the scalpel before and after they are in contact with the fungus following proper aseptic techniques.
2 Add 400 μl TE buffer containing RNase to the sample.
  Work should be performed in a circulating Class II flow hood near a Bunsen burner.
3 Grind the mycelium in a Geno/Grinder for 2 min at 17,700 × *g*, room temperature.
  Work is performed at room temperature.
4 Centrifuge 10 min at 14,000 × *g*, room temperature.
5 Transfer 100 μl of the supernatant to a fresh 1.5‐ml microcentrifuge tube, using 1‐5 μl per eventual PCR reaction. Sample can be stored in a freezer set at −20°C for future uses.
  Usually, 1 μl of the obtained supernatant should be enough to perform one PCR reaction. This volume will depend on the size of mycelium cut for the extraction. Concentration is usually measured using a NanoDrop spectrophotometer and samples stored at −20°C. The homogenizer metal bead can be cleaned, sterilized, and reused.

## Basic Protocol 4 *M. ORYZAE* INDUCTION OF APPRESSORIUM DEVELOPMENT ON GLASS COVERSLIPS FOR MICROSCOPY

**Figure 3:**
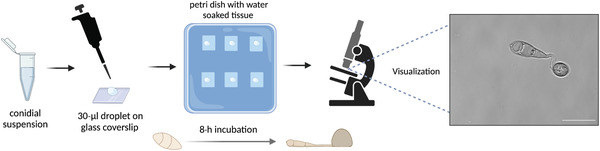
Schematic representation of the induction of *M. oryzae* appressorium development on hydrophobic coverslips. 30‐μl droplets of conidial suspension (5 × 10^4^ conidia/ml) are inoculated on hydrophobic coverslips and incubated from 2 hr up to 24 hr on square petri dishes containing water‐soaked tissue paper to create a humid environment; they are later visualized by microscopy. On the right, a typical microscopy image (×100) of germinated appressoria (of strain Guy11) on coverslips after 8 hr of inoculation. Scale bar, 10 μm. Figure created with BioRender (biorender.com).

In order to study *M. oryzae* appressorium development, methods to induce conidial germination and induction of appressorium formation have been developed in the laboratory. In vitro appressorium formation was initially shown to be possible on hard surfaces, such as a coverslip, in 1906 (Hasselbring, 1906). Surface hydrophobicity was later shown to be a crucial determinant for appressorium development (Lee & Dean, 1994), and hard surfaces normally need to have contact angles with water of >90^o^ to stimulate appressorium formation (Talbot, Ebbole, & Hamer, 1993). For this reason, appressorium formation has been routinely carried out on polytetrafluoroethylene (PTFE, Teflon) surfaces or GelBond film, but it can also be stimulated on some types of glass coverslips with sufficient surface hydrophobicity, which have better optical properties for microscopy than PTFE. Currently, with specific hydrophobic coverslips and the right humidity settings, appressorium formation and maturation is easily achieved in the laboratory (Fig. 3). This protocol describes how to achieve conidial germination in the laboratory for the visualization of appressorium development by light microscopy.

*CAUTION: Magnaporthe oryzae* is a Biosafety Level 1 pathogen; follow all appropriate guidelines and regulations for the use and handling of pathogenic microorganisms.

### Materials

70% (v/v) ethanol (in water) for decontaminating materials and equipment
7‐ to 10‐day‐old *M. oryzae* fungal culture grown on CM agar medium (Basic Protocol 2 or Alternate Protocol 1)
Sterile distilled water

Circulating Class II flow hood (BSC Class II or equivalent)
Bunsen burner (e.g., Fisher Scientific cat. no. 03391301)
L‐shaped cell spreaders (e.g., Fisherbrand™ cat. no. 15615467)
Filter cloth or polypropylene membrane (e.g., Sigma cat. no. Z104256), sterile
50‐ml conical Falcon tubes (e.g., Fisher Scientific cat. no. 10788561)
1.5‐ml microcentrifuge tubes (e.g., Eppendorf ® Microtube capacity 1.5 ml cat. no. EP0030125150)
Filter tips, volume 20‐200 μl and volume 100‐1000 μl (e.g., Eppendorf cat no s. EP3123000055 and EP3123000063)
Single‐channel micropipets, volume 20‐200 μl and volume 100‐1000 μl (e.g., Eppendorf cat. nos. EP3123000241 and EP3123000250)
Microtube centrifuge (e.g., Fisher Scientific cat. no. 75002435)
Hemocytometer (e.g., Sigma cat. no. Z359629‐1EA)
Biological microscope (e.g., Fisherbrand™ cat. no. 15374398)
120‐mm square plastic petri dish (e.g., Scientific Laboratory Supplies cat. no. PET3008)
Paper towel (e.g., DIY)
Hydrophobic coverslips (Eprendia coverslips 18 × 18 mm #2)
Incubator (e.g., Fisher Scientific cat. no. 15500214), set at 24°C

1 Pour ∼3 ml sterile distilled water onto a 7‐ to 10‐day‐old *M. oryzae* culture grown on CM agar medium and gently harvest mycelium with an L‐shaped cell spreader to release fungal conidia.
  Work should be performed in a circulating Class II flow hood near a Bunsen burner. Use 5 ml of water for cultures that sporulate in abundance and 1.5‐2 ml of water for cultures with deficient sporulation.
2 Place a double‐folded sterile filter cloth on a 50‐ml Falcon tube and filter the liquid containing fungal mycelium and conidia.
  After filtering, squeeze filter cloth, enabling extra conidia to filter through.
3 Transfer 1.5 ml of the filtered liquid to a 1.5‐ml microcentrifuge tube using a micropipet.
  If filtered liquid is very light colored, indicating low abundance of conidia, repeat step 3 to obtain a visible pellet of conidial cells after centrifugation in step 4.
4 Centrifuge 5 min at 8000 × *g*, room temperature.
5 Discard the supernatant and resuspend pellet (conidia) in 1000 μl sterile distilled water.
6 Count conidial cells using a hemocytometer and a biological microscope, and adjust conidial suspension to a concentration of 5 × 10^4^ conidia/ml.
  For microscopy purposes, prepare ∼100 μl of conidial suspension.
7 Prepare square plastic petri dishes with two layers of paper soaked in sterile distilled water and place hydrophobic coverslips on top (see Fig. 3).
  Make sure paper towels have enough water to be completely soaked, but pour away any water that has not been absorbed by paper towel, as it can prevent germination. Usually, three cover slips per sample are used.
8 Pipet 30‐μl droplet of conidial suspension onto each coverslip, place the lid on the plastic dish, and incubate at 24°C for appressorium induction.
  Be careful so droplets do not lose shape during incubation. Appressoria will be visible from 2 hr after spore inoculation and will continue to mature for up to 24 hr before collapsing.
9 To visualize under the microscope, carefully collect coverslip after the desired incubation and place it on a microscopy slide. Dry out excess water and visualize.

## Alternate Protocol 3 *M. ORYZAE* INDUCTION OF APPRESSORIUM DEVELOPMENT ON GLASS COVERSLIPS FOR TISSUE COLLECTION

For tissue collection from appressoria, experiments must be performed at a larger scale than described in Basic Protocol 4. This protocol explains how to achieve large amounts of conidial germination in the laboratory using hydrophobic coverslips mounted onto petri dishes. Commonly, appressorial tissue collection is performed to extract genomic DNA, RNA, or protein from the fungal material.

*CAUTION: Magnaporthe oryzae* is a Biosafety Level 1 pathogen; follow all appropriate guidelines and regulations for the use and handling of pathogenic microorganisms.

### Additional Materials (also see Basic Protocol 4)

Superglue (e.g., Loctite)
1,16‐Hexadecanediol

Circulating Class II flow hood (BSC Class II or equivalent)
Bunsen burner (e.g., Fisher Scientific cat. no. 03391301)
120‐mm square plastic petri dish (e.g., Scientific Laboratory Supplies cat. no. PET3008)
Hydrophobic coverslips (Eprendia coverslips, 18 × 18 mm, #2)
1.5‐ml microcentrifuge tubes (e.g., Eppendorf ® Microtube capacity 1.5 ml cat. no. EP0030125150)
Single‐channel micropipets, volume 20‐200 μl and volume 100‐1000 μl (e.g., Eppendorf cat. nos. EP3123000241 and EP3123000250)
Filter tips, volume 20‐200 μl and volume 100‐1000 μl (e.g., Eppendorf cat. nos. EP3123000055 and EP3123000063)
50‐ml conical Falcon tubes (e.g., Fisher Scientific cat. no. 10788561)
Incubator (e.g., Fisher Scientific cat. no. 15500214), set at 24°C
Steel razor blades (e.g., Fisher Scientific cat. no. 11904325)
Liquid nitrogen
Freeze dryer (e.g., Labconco™ cat. no. 16212551)
Ultra‐low freezer (e.g., Fisherbrand™ cat. no. 17426432), set to −80°C

1 Prepare as much volume as possible of conidial suspension adjusted to 5 × 10^4^ conidia/ml following steps 1‐6 of Basic Protocol 4 (50 ml of conidial suspension will be used per square petri dish, and usually 2‐5 petri dishes will be used per sample).
  Work should be performed in a circulating Class II flow hood a Bunsen burner. Use as many M. oryzae culture plates grown on CM as needed to obtain the desired volume of conidial suspension. Occasionally 50 plates are harvested to achieve the desired amount of conidia in the laboratory.
2 Prepare 120‐mm square plastic petri dishes with hydrophobic coverslips glued to the bottom of each dish using Superglue.
  Preparing the plastic petri dishes with glued coverslips before starting the experiment is recommended. Use very small amounts of glue, as this can disturb appressorium germination. Attach as many coverslips as will fit to cover the bottom surface of the dish; this will depend on the petri dish being used, but we usually attach eight per dish.
3 Add 2.5 mg/ml of 1,16‐hexadecanediol per 50 ml of conidial suspension.
  1,16‐Hexadecanediol will stimulate appressorium germination to occur synchronously, preventing collection of tissue at different germination stages.
4 Pour 50 ml of conidial suspension into each petri dish, place the lid on each dish, and incubate at 24°C to allow appressorium induction.
  Germination timepoints typically used range from 0 hr to 24 hr. At later timepoints, appressoria formed in coverslips will collapse.
5 After desired incubation period, pour away liquid inside petri dish and, using a steel razor blade, scrape off the appressoria that have attached and germinated on the coverslips
  Perform this step quickly to achieve uniformity in appressorium development of different samples.
6 Collect the material into a clean 50‐ml Falcon tube and immediately freeze using liquid nitrogen.
  You can obtain ∼50 mg of appressorial tissue per square petri dish.
7 Lyophilize samples using a freeze‐drier for 24 hr. After this, samples can be used immediately or kept in a freezer at −80°C until required.
  Samples are frozen using liquid nitrogen. However, there is usually a very small amount of material, and removing liquid as much as possible will enhance the following extractions.

## Basic Protocol 5 *M. ORYZAE* RICE INFECTION ASSAY THROUGH SPRAY INOCULATION

Spray inoculation pathogenicity assays are commonly used to test the ability of different *M. oryzae* strains to cause blast disease. In this protocol we explain how we currently use fungal conidial suspensions to spray‐inoculate rice seedlings, how to incubate them, and how to later evaluate disease symptoms. This is based on one of the first published protocols for infecting rice seedlings (Talbot et al., 1993; Valent & Chumley, 1991).

*CAUTION: Magnaporthe oryzae* is a Biosafety Level 1 pathogen; follow all appropriate guidelines and regulations for the use and handling of pathogenic microorganisms.

### Materials

70% (v/v) ethanol (in water) for decontaminating materials and equipment
7‐ to 10‐day‐old *M. oryzae* fungal culture grown on CM agar medium (Basic Protocol 2 or Alternate Protocol 1)
0.1% (w/v) gelatin
3‐week‐old blast‐susceptible rice (*Oryza sativa*) cultivar (such as CO‐39, Kitaake, or Nipponbare) grown in 9‐cm plastic pots/trays

Circulating Class II flow hood (BSC Class II or equivalent)
Bunsen burner (e.g., Fisher Scientific cat. no. 03391301)
L‐shaped cell spreaders (e.g., Fisherbrand™ cat. no. 15615467)
50‐ml conical Falcon tubes (e.g., Fisher Scientific cat. no. 10788561)
Filter cloth or polypropylene membrane (e.g., Sigma cat. no. Z104256)
Filter tips, volume 20‐200 μl and volume 100‐1000 μl (e.g., Eppendorf cat. nos. EP3123000055 and EP3123000063)
1.5‐ml microcentrifuge tubes (e.g., Eppendorf ® Microtube capacity 1.5 ml, cat. no. EP0030125150)
Single‐channel micropipets, volume 20‐200 μl and volume 100‐1000 μl (e.g., Eppendorf cat. nos. EP3123000241 and EP3123000250)
Microtube centrifuge (e.g., Fisher Scientific cat. no. 75002435)
Hemocytometer (e.g., Sigma cat. no. Z359629‐1EA)
Biological microscope (e.g., Fisherbrand™ cat. no. 15374398)
120‐mm square plastic petri dish (e.g., Scientific Laboratory Supplies cat. no. PET3008)
Mini airbrush sprayer (e.g., Badger USA)
Polyethylene plastic transparent bags, ∼229 mm × 305 mm (e.g., Polybags)
Controlled‐environment chamber (e.g., Memmert™ cat. no. 15607578), set to 24°C with a 12‐hr photoperiod and 85% relative humidity
Whatman filter paper (e.g., Fisherbrand™ cat. no. 09800)

**Figure 4:**
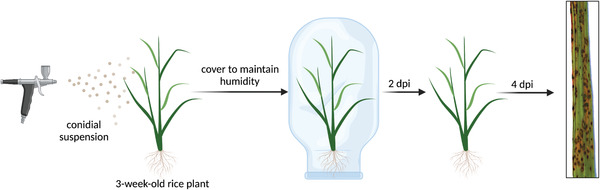
Schematic representation of *M. oryzae* spray‐inoculation protocol. Conidial suspensions are evenly sprayed onto 3‐week‐old rice (CO‐39) seedlings using an artist's airbrush. Plants are then incubated for 6 days (2 days covered with polyethylene bag and 4 days uncovered) in a controlled chamber set at 24°C with a 12‐hr photoperiod and 85% relative humidity. At right, an example of CO‐39‐infected leaf tip infected with *M. oryzae* Guy11 spores 6 days after spray inoculation. Figure created with BioRender.com.

1 Follow steps 1‐6 of Basic Protocol 4 to obtain conidial suspension adjusted to 5 × 10^4^ conidia/ml in 0.1% (w/v) gelatin.
  Work should be performed in a circulating Class II flow hood near a Bunsen burner. Calculate 5 ml of conidial suspension to be sprayed over each rice plant.
2 Using an airbrush, spray‐inoculate ∼5 ml of conidial suspension onto 3‐week‐old blast‐susceptible rice (*Oryza sativa*) cultivar CO‐39 plants, and cover plants with polyethylene bags (see Fig. 4).
  Perform spraying in controlled conditions, preventing any sort of leakage.

3 In a controlled‐environment chamber at 24°C with a 12‐hr photoperiod and 85% relative humidity, incubate rice plants for 48 hr covered with the plastic bag and then for 4 more days uncovered.
4 After incubation, randomly collect infected leaf tips (∼5 cm) and attach them to Whatman paper using adhesive tape at each end to secure to the surface.
5 Assess leaf infection density by calculating the mean number of disease lesions from a ∼2‐cm infected area of leaf. Significant differences between leaf infection density are later calculated by performing statistical comparisons.
  Counting of the lesions should be performed in 1.5‐3 cm of the most densely infected area. Lesions usually look like dark brown dots with lighter surrounding margins. Counting is typically performed manually, although methods for automated assessment of leaf infection using image analysis are under development.

## Alternate Protocol 4 *M. ORYZAE* LEAF‐DROP PLANT INFECTION ASSAY

To rapidly evaluate the virulence of *M. oryzae* strains, leaf‐drop inoculation is routinely used. This provides a qualitative evaluation of the ability to cause blast disease, but this can be made quantitative by measuring fungal biomass using a DNA‐based assay. Using this protocol, non‐pathogenic strains of *M. oryzae* can be easily differentiated from pathogenic isolates.

*CAUTION: Magnaporthe oryzae* is a Biosafety Level 1 pathogen; follow all appropriate guidelines and regulations for the use and handling of pathogenic microorganisms.

### Additional Materials (also see Basic Protocol 4)

0.2% (w/v) gelatin
3‐week‐old blast‐susceptible rice (*Oryza sativa*) cultivar (CO‐39) or 1‐week‐old barley cultivar (Golden Promise)
4% (w/v) distilled water agar plated in 9‐cm petri dishes

Incubator (e.g., Fisher Scientific cat. no. 15500214), set at 24°C with a 12‐hr photoperiod
120‐mm square petri dishes (e.g., Scientific Laboratory Supplies cat. no. PET3008)
Whatman filter paper (e.g., Fisherbrand™ cat. no. 09800)

**Figure 5:**
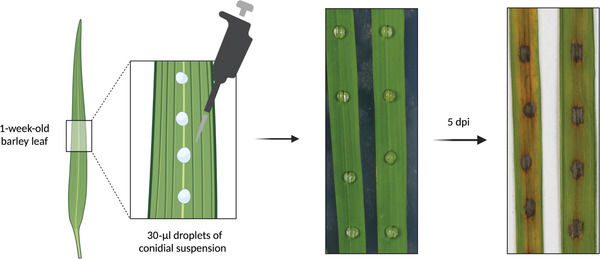
*M. oryzae* leaf‐drop plant infection assay using 1‐week‐old barley (cultivar Golden Promise). Left, Schematic representation. Right, barley leaf fragments (∼4 cm) inoculated with 30‐μl droplets of Guy11 conidial suspension (5 × 10^4^ conidia/ml) and incubated at 24°C with a 12‐hr photoperiod, before and 5 days after inoculation. Figure created with BioRender (biorender.com).

1 Follow steps 1‐6 of Basic Protocol 4 to obtain conidial suspension adjusted to 5 × 10^4^ conidia/ml, and add 0.2% (v/v) gelatin.
  Work should be performed in a circulating Class II flow hood near a Bunsen burner. Calculate ∼50 μl of conidial suspension to be used, for 10‐ to 15‐μl droplets inoculated on three or four different leaves.
2 Cut three or four 5‐cm same‐age leaf tips (either 3‐week‐old CO‐39 or 1‐week‐old barley Golden Promise) and place on a square petri dish containing 4% water agar as shown in Figure 5.
  Golden Promise leaf tips are wider, making this experiment easier when using barley leaves than when using rice leaves, because the conidial suspension droplet does not slide off the leaf (due to surface hydrophobicity) as easily.

3 Inoculate 10‐ to 15‐μl droplets of conidial suspension from each sample on each leaf.
  Make sure to use several leaf tips and several points for inoculation of the same sample to achieve robust and replicated results.
4 Incubate leaf tips 5 days at 24°C with a 12‐hr photoperiod.
  Susceptible infections can be visualized earlier than 5 days, but it is advisable to leave for sufficient time to avoid false negative results.
5 After incubation, collect leaf tips and mount on Whatman paper to visually assess blast symptoms.
  Usually leaf‐drop infection experiments are used to evaluate resistant or non‐resistant plants or virulent or non‐virulent mutants of the fungus. However, lesions can be harvested and transferred to a 2‐ml tube with 300 μl of distilled water, spores recovered through mixing with a vortex, and the conidial concentration quantified using a hemocytometer.

## REAGENTS AND SOLUTIONS

### Complete medium (CM) (Talbot et al., 1993)

1 g/L casamino acids (e.g., Sigma‐Aldrich cat. no. 2240OP)
10 g/L glucose (e.g., Sigma‐Aldrich cat. no. G8270)
2 g/L peptone (e.g., Sigma‐Aldrich cat. no. 83059)
1 g/L yeast extract (e.g., DB Bioscience)
0.1% (v/v) vitamin supplement:
  0.001 g/L biotin (e.g., Sigma‐Aldrich cat. no. B4501)
  0.001 g/L pyridoxine (e.g., Sigma‐Aldrich cat. no. P5669)
  0.001 g/L thiamine (e.g., Sigma‐Aldrich cat. no. T4625)
  0.001 g/L riboflavin (e.g., Sigma‐Aldrich cat. no. 1.07609)
  0.001 g/L nicotinic acid (e.g., Sigma‐Aldrich cat. no. 170860)
6 g/L NaNO_3_ (e.g., Sigma‐Aldrich cat. no. S5506)
0.5 g/L KCl (e.g., Sigma‐Aldrich cat. no. 529552)
0.5 g/L MgSO_4_ (e.g., Sigma‐Aldrich cat. no. M7506)
1.5 g/L KH_2_PO_4_ (e.g., Sigma‐Aldrich cat. no. P0662); bring to pH 6.5 with NaOH
 0.1% (v/v) trace elements:
  22 mg/L zinc sulfate heptahydrate (e.g., Sigma‐Aldrich cat. no. 221376)
  11 mg/L boric acid (e.g., Sigma‐Aldrich cat. no. B0394)
  5 mg/L manganese(II) chloride tetrahydrate (e.g., Sigma‐Aldrich cat. no. M3634)
  5 mg/L iron(II) sulfate heptahydrate (e.g., Sigma‐Aldrich cat. no. 215422)
  1.7 mg/L cobalt(II) chloride hexahydrate (e.g., Sigma‐Aldrich cat. no. 255599)
  1.6 mg/L copper(II) sulfate pentahydrate (e.g., Sigma‐Aldrich cat. no. 1.02780)
  1.5 mg/L sodium molybdate dehydrate (e.g., Sigma‐Aldrich cat. no. M1003)
  50 mg/L ethylenediamine tetraacetic acid (EDTA; e.g., Sigma‐Aldrich cat. no. 324503)
15 g/L agar (for solid medium; e.g., Sigma‐Aldrich cat. no. A1296)
Autoclave the medium according to normal procedures (121°C for 60 min), let cool to 50°C, add Penicillin‐Streptomycin (50 U/ml; e.g., Fisher Scientific cat. no. 15140122), and then plate on 9‐cm petri dishes. The plates can be stored at 4°C up to 4 weeks.

### Sodium acetate (pH 5.2), 3 M

24.6 g sodium acetate (anhydrous; e.g., Sigma‐Aldrich cat. no. W302406)
100 ml sterile distilled water

### TE buffer, 1×

10 mM Tris, pH adjusted to 8.0 with HCl (e.g., Sigma‐Aldrich cat. no. T1699)
1 mM EDTA, pH adjusted to 8.0 with NaOH (e.g., Sigma‐Aldrich cat. no. E9884)

## COMMENTARY

### Background Information

The first experiments performed using *M. oryzae* isolates were the evaluation of pathogenicity and simple growth assays (Trevathan, 1982). After this, classical genetic analysis was achieved in *M. oryzae* because it is a heterothallic fungus with two different mating types, *MAT1‐1* and *MAT1‐2*, that when paired together enable the fungus to undergo sexual reproduction, producing perithecia from which eight‐spored asci can be harvested and ascospores collected for octad analysis and random ascospore analysis (Valent & Chumley, 1991). One of the *M. oryzae* rice pathogenic strains now widely used in most laboratories, discovered in French Guiana and called Guy11, was originally isolated because of its fertility. As a consequence, it has been extensively used since the late 1980s for sexual crossing, and many mutants have been generated in the Guy11 strain background (Leung, Borromeo, Bernardo, & Notteghem, 1988; Silué & Notteghem, 1992).

Long‐term preservation of fungal strains aims to maintain cultures without causing any genetic or physiological changes (Smith & Ryan, 2012). Given that cryopreservation is extensively used for storage in microbiology, it was the first method by which filamentous fungi were preserved (Guo, Wei, & Xu, 2020; Smith, 1988). However, water crystallization of cultures can be problematic for filamentous fungi, and therefore lyophilization became the preferred method in many species, including *M. oryzae* (Tan, van Ingen, & Stalpers, 2007). Other procedures, such as preservation in distilled water or on cotton wool buds, have also been used for semi‐long‐term storage of filamentous fungi (∼1 year; Al‐Bedak, Sayed, & Hassan, 2019; Bueno & Gallardo, 1998). Nevertheless, the dry filter paper technique described here has proven to be the best method to preserve *M. oryzae* for long periods and is ideally suited to the rice blast fungus because of its mimicry of the natural overwintering behavior of the fungus. It is very simple to perform, and preserving frozen strains at −20°C rather than as glycerol stocks at −80°C (Fong et al., 2000) requires less energy and far less space, given that many hundreds of filter paper stocks can be stored in an individual paper bag, with many thousands of isolates then stored in a single plastic storage box. Maintenance and growth techniques for the wheat blast fungus have been described recently (Gupta et al., 2020). Potato dextrose agar (PDA) and oatmeal agar (OMA) were the first growth media reported for *M. oryzae* growth (Crawford, Chumley, Weaver, & Valent, 1986; Trevathan, 1982). They are still frequently used, but for many years *M. oryzae* laboratory strains derived from Guy11 have been successfully grown on CM medium, which has the advantage of being a defined growth medium that does not suffer from batch‐to‐batch variation (Talbot et al., 1993). In our laboratory, CM has been the regular growth medium and supports growth and conidiation very efficiency at 24°C with a 12‐hr photoperiod.

In vitro appressorium induction has been successfully carried out for a long time for a small number of fungal pathogens using hard surfaces such as coverslips (Hasselbring, 1906). For *M. oryzae*, appressorium development was once routinely carried out using PTFE (Teflon) membranes (Hamer et al., 1988), but this has been superseded because of the superior optical qualities of hydrophobic glass coverslips. On hydrophilic surfaces, *M. oryzae* conidia germinate to produce long germ tubes that do not form appressoria. To overcome this problem, high concentrations of pharmacologic agents that stimulate cyclic AMP signaling were initially used to induce appressorium development (Lee & Dean, 1993). However, appressorium induction protocols have now been developed, using hydrophobic glass surfaces and the correct humidity settings, that achieve efficient *M. oryzae* conidial germination and appressorium development without any external inducer (Lee & Dean, 1994). The addition of hexadecanediol, a cutin monomer that mimics the rice leaf surface, does, however, stimulate more synchronous appressorium development, which is valuable for producing large numbers of infection cells for DNA, RNA, or protein extraction.

Good‐quality fungal DNA extractions have been possible since the early 1990s (Rogers & Bendich, 1994). These protocols have not evolved significantly, but it is now easier to extract high‐quality genomic DNA with the protocol described here. The same applies to infection assays, both spray and leaf‐drop inoculations, which now produce highly reproducible disease symptoms (Valent et al., 1991; Xie & Mew, 1998).

### Critical Parameters and Troubleshooting

#### M. oryzae storage on desiccated filter paper

Before freezing *M. oryzae* filter stocks, make sure they are properly desiccated because any humidity/water can crystallize, causing modifications to the DNA or the physiology of the strain (Tan et al., 2007). Additionally, if filter stocks are not prepared or kept properly, they will lose viability. It is advised that humidified silica beads be replaced frequently (at least once per month) even when kept at −20°C.

#### M. oryzae growth on solid and liquid CM

*M. oryzae* growth is significantly affected by temperature, decreasing below 15°C or above 38°C (Gupta et al., 2020). 24°C is the usual *M. oryzae* growth temperature to obtain healthy mycelium. Growing below or above this temperature will affect growth and may stress the culture. Moreover, when growing *M. oryzae* in liquid CM, the rotary shaker must be carefully set to 100 rpm, as shaking below or above this speed will affect growth or even prevent growth.

Common contaminants of fungal cultures are laboratory bacteria such as *Escherichia coli*, *Agrobacterium tumefaciens*, and *Streptomyces*, as well as aerial fungal contaminants such as *Cladosporium* and *Penicillium* species. When cultures are contaminated, mycelium growth may be suppressed (Li et al., 2011). Avoid contamination by working with strict aseptic technique in a circulating airflow cabinet (class II). It is also advisable to use UV light for decontamination before and after working with *M. oryzae*.

#### M. oryzae mycelium DNA extraction

Extraction of good‐quality genomic DNA from *M. oryzae* mycelium is usually achievable, but there are a few common problems, which can be solved using the following troubleshooting guide (see Table 1).

**Table 1:** . Troubleshooting Guide for DNA Extraction From *M. oryzae* Fungal Mycelium

Problem	Possible cause	Solution
Low concentration of DNA	Mycelium not ground vigorously or thoroughly enough	Make sure the powder obtained after grinding in pestle and mortar is extremely fine.
	CTAB buffer was not preheated to 65°C	Preheat CTAB buffer at 65°C before mixing it with fungal material.
	Not enough or too much CTAB buffer used	Use an equal amount of CTAB buffer and fungal material (v/w) for the extraction.
	Not sufficiently precipitated	Make sure precipitation steps are long enough, increasing all of them up to 24 hr.
Poor quality of DNA	Aqueous supernatant not collected properly	Meticulously collect supernatant after incubation with CIA to avoid contamination of DNA.
	Washes performed too fast	Make sure to wash DNA properly with 70% ethanol.
	Propanol/ethanol remaining after draining	Make sure there is no liquid on the tube after draining; this can also be done using a SpeedVac vacuum concentrator.

#### Appressorium induction on coverslips

*M. oryzae* appressorium development can be easily accomplished in the laboratory. However, there are a few critical factors to take into consideration. First, hydrophobicity and cell starvation are known to be key inducers of appressorium development (Talbot, 2003), so it is important to use the correct hydrophobic coverslips to achieve appressorium formation. Secondly, conidial suspensions should not have a concentration above 5 × 10^4^ conidia/ml, because a higher appressorial density inhibits germination, possibly through release of fungal self‐inhibitory molecules. Finally, working in a circulating airflow cabinet (class II) is recommended to avoid contamination of the conidial suspension with bacteria, which can inhibit *M. oryzae* germination (Li et al., 2011).

#### M. oryzae infection assays

When performing infection assays, the main consideration is to ensure the plants are healthy and well‐fertilized before use. Wounded or stressed plants will result in altered infection results and should never be used. Moreover, conidial suspensions should always have the same concentration to enable comparison of results. Densities of >5 × 10^4^ conidia/ml must be avoided, as stated above, as *M. oryzae* can self‐inhibit germination when conidial suspensions exceed 1 × 10^5^ conidia/ml.

### Understanding Results

When visualizing appressoria under a microscope, depending on the time‐point of conidial germination or appressorium development, distinct developmental stages can be identified. During the first 2 hr of germination, the germ tube will emerge and grow from the apical cell of the conidium (Wilson & Talbot, 2009). Sometimes, on coverslips, these germ tubes can elongate more than expected, possibly searching for correct developmental conditions. After this, the tip of the germ tube swells and forms a new cell structure, the appressorium (Bourett & Howard, 1990). During later time‐points (4‐24 hr), appressoria will mature by generating melanin that localizes between the appressorium plasma membrane and the cell wall (Bourett & Howard, 1990). This layer of melanin can be visualized under a microscope as it becomes darker due to melanin deposition. At later time‐points, appressoria appear heavily melanized and also increase in size during turgor generation (de Jong et al., 1997).

Regarding infection assays, it is important to distinguish disease lesions on susceptible hosts from the hypersensitive response (HR), which occurs on resistant hosts as a defense mechanism or when a plant has been exposed to other stresses (Balint‐Kurti, 2019; Jones & Dangl, 2006). Generally, disease lesions will appear darker brown/gray colored surrounded by a lighter yellow/light brown halo, and they will continue to grow over time. On the other hand, resistant HR response will be clearly lighter in color, usually yellow or light brown, and will not grow over time. It is important to carefully assess disease lesions, avoiding misperception.

### Time Considerations

For every protocol described, *M. oryzae* is pregrown on CM agar medium. This usually takes between 7 and 10 days. We recommend strategic planning of fungal growth before performing any of the explained protocols.

Fungal DNA extraction can be performed in ∼4 hr. However, longer precipitation of DNA, for example overnight at −20°C, will increase the efficiency of the extraction.

### Author Contributions

**Camilla** Molinari: Conceptualization, investigation, methodology, writing—original draft; **Nicholas J**. Talbot: Conceptualization, funding acquisition, methodology, writing—original draft.

### Conflict of Interest

Authors declare no conflict of interest.

## Data Availability

The data that support the findings of this study are available from the corresponding author upon reasonable request
